# Immunohistochemical-properties of the dermal embryonic telocytes

**DOI:** 10.1038/s41598-024-63802-5

**Published:** 2024-06-17

**Authors:** Soha A. Soliman

**Affiliations:** Department of Histology, Faculty of Veterinary Medicine, South Valley University, Qena, Egypt

**Keywords:** Telocytes, Dermis, CD34, CD117, VEGF, MMP-9, CD68, CD21, Cell biology, Anatomy

## Abstract

The current investigation aims to study the embryonic dermis formed in the early stages of development and identify the initial interstitial components of the dermis that serve as biological and structural scaffolds for the development of the dermal tissue. To investigate the dermal structure, the current study used morphological and immunological techniques. TCs identified by TEM. They had a cell body and unique podomeres and podoms. They formed a 3D network spread throughout the dermis. Homocellular contact established between them, as well as heterocellular contacts with other cells. Immunohistochemical techniques using specific markers for TCss CD34, CD117, and VEGF confirmed TC identification. TCs represent the major interstitial component in the dermal tissue. They established a 3D network, enclosing other cells and structures. Expression of VEGF by TC promotes angiogenesis. TCs establish cellular contact with sprouting endothelial cells. At the site of cell junction with TCs, cytoskeletal filaments identified and observed to form the pseudopodium core that projects from endothelial cells. TCs had proteolytic properties that expressed MMP-9, CD68, and CD21. Proteolytic activity aids in the removal of components of the extracellular matrix and the phagocytosis of degraded remnants to create spaces to facilitate the development of new dermal structures. In conclusion, TCs organized the scaffold for the development of future dermal structures, including fibrous components and skin appendages. Studying dermal TCs would be interested in the possibility of developing therapeutic strategies for treating different skin disorders and diseases.

## Introduction

Telocytes are interstitial cells that form a network communicating system between diverse types of cells, either resident or non-resident***.*** The structural profile of telocytes enables cellular contact through their telopodes, or cell prolongations, which can grow to be hundreds of microns long. Telopodes are made up of dilated segments called podoms and thin segments called podomers, where the endoplasmic reticulum and mitochondria are grouped together^1,2^.

Based on gene expression data, telocyte function in cell signaling^3,4^; tissue homeostasis, repair, remodeling, and angiogenesis^5^; suppression of oxidative stress and cellular aging^6^; and a protective role against inflammatory processes and oncogenesis^7^**.**

Cell-to-cell communication is one of the primary properties of telocytes. Both cell contact and the paracrine route are considered as channels for telocyte communication. Several types of cell contact between telocytes and other cells have been described, including cell contact at intermembrane distances where macromolecules interact and minute junctions, such as point contacts, nanocontacts, and planar contacts^8^. Telocytes can form a variety of cell contacts, including gap junctions, Puncta Adherens minima, and Adherens. A gap junction enables the transfer of signals between cells^8,9^. Telocytes transport active molecules to neighboring cells by a paracrine signaling pathway through secretory vesicles, exosomes, ectosomes, and multivesicular vesicles^1,10, 11^.

The Japanese quail (Coturnix japonica) is widely used as a lab animal for biological purposes, including immunology, endocrinology, aging, developmental biology, behavior studies, and a range of hereditary abnormalities in humans^12,13^. Japanese quail (Coturnix coturnix japonica) is frequently used in research as an accessible model to study. This study is a morphological study that serves as a foundation to investigate the developmental events that occur during embryonic stages, providing a clear view for further investigations that integrate genomic and morphological data. Much of our knowledge about developmental biology and functional genomics has come from the study of avian embryology. Quail have a significant advantage as a favorable model for transgenic birds, with recent years seeing the description of transgenic quail lines. However, most of these were generated using replication-deficient lentiviruses, a technique that presents various limitations. A new technology has been developed for quail transgenesis that is based on the in vivo transfection of plasmids in circulating Primordial Germ Cells (PGCs). This technique is simple, efficient, and allows for the use of the infinite variety of genome engineering approaches developed in other models^14–17^.

The current investigation focused on the structure of the primitive dermis at an early stage of development before the distinctive formation of fibrous contents and identifying their components, which comprise the infrastructure of the future dermis. TCs are a unique type of interstitial cell that predominates the interstitium of embryonic tissue (unpublished data^18,19^). The present research aims to identify the early structure of the embryonic dermis formed before building up the dermal components, such as fibrous components and skin appendages. The early structures serve as the biological and structural scaffold for the development of other dermal components. Studying dermal TCs in detail may shed light on the skin's innate ability to repair itself. Furthermore, it would be appealing to think about the possibility that increasing the density or presence of dermal TCs could end up being a very appealing therapeutic option for treating different skin conditions.

## Results

The current study aimed to identify dermal telocytes during the early stages of embryonic development using histochemistry, TEM, and IHC.

### Microscopic dermal structures of day 8 embryos using H&E

**Figure 1:**
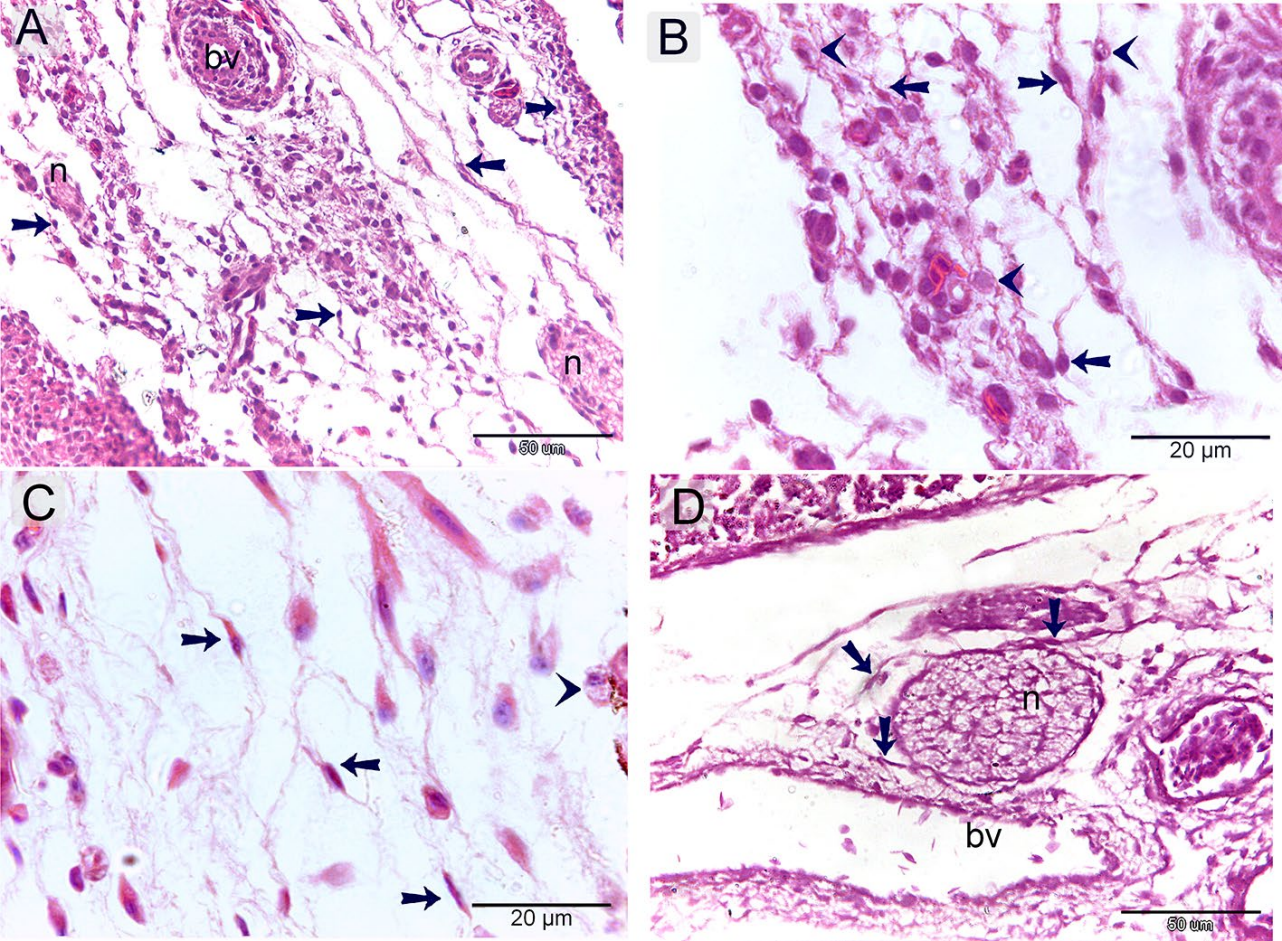
Microscopic dermal structures of day 8 embryos using H&E. Paraffin sections of skin embryos stained by H&E (**A**–**C**) and PAS (**D**). (**A**–**C**): the dermis formed of a loose connective tissue that developed blood vessels (bv) and nerve fibers (n). The majority of interstitial cells (arrows) had a cell body and cellular prolongations. Other cells had vacuolated cytoplasm (arrowheads). (**D**): the dermic contained growing nerve fibers (n) that had limited PAS developing myelin sheath. Note blood vessels (bv).

At day 8 of incubation, the dermis developed loosely arranged interstitial elements that contained blood vessels, nerves, and immune cells (Fig. 1A–D). The majority of interstitial cells had a cell body and cellular prolongations (Fig. 1C).

### Microscopic dermal structures of day 8 embryos using toluidine blue

**Figure 2:**
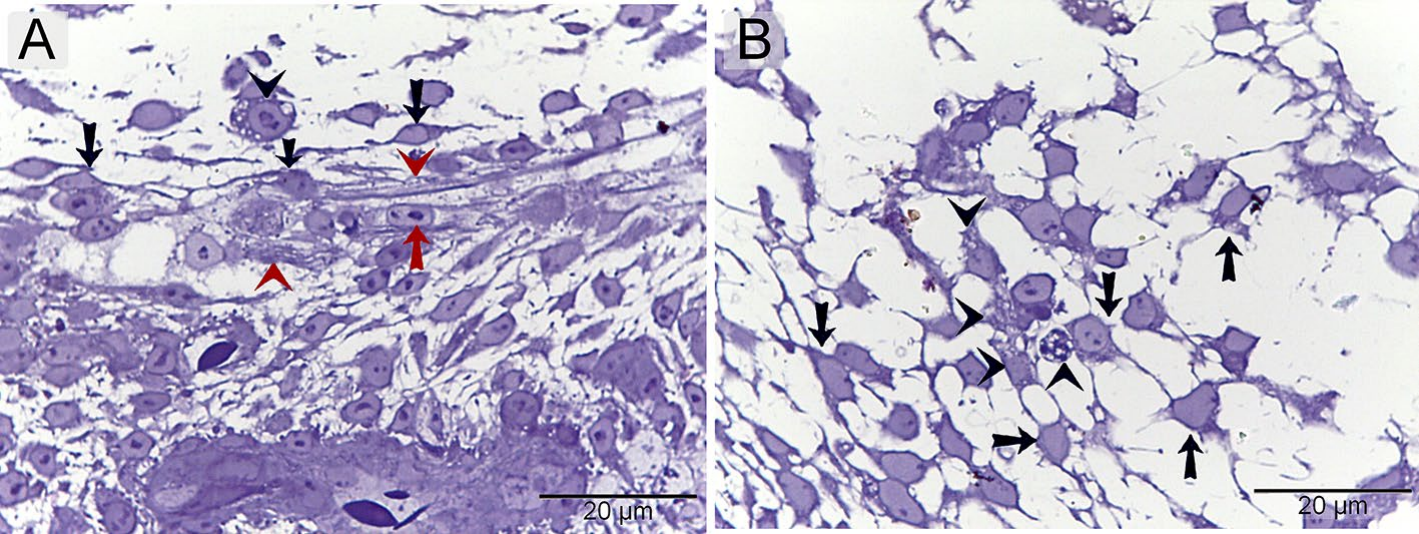
Microscopic dermal structures of day 8 embryos using toluidine blue. Semithin section of skin embryos stained by toluidine blue. (**A**): the dermis formed of a loose connective tissue that continued developing nerve fibers. Multilayered myelin membrane of Schwann cell (red arrowheads), Schwann cell (red arrow), TCs (Telocytes) (black arrows) around the Schwann cell at the area of myelin sheath formation. (**B**): The dominant type of interstitial cells (arrows) seems to be TCs that had a cell body and cellular prolongations. Macrophage had vacuolated cytoplasm (arrowheads).

The dermis was formed of loose connective tissue that contained developing nerve fibers. TCs were identified around the multilayered myelin membrane of Schwann cells, where myelin sheath formation occurred (Fig. 2A). TCs were the principal interstitial cells that were recognized by the cell body and prolongations. Macrophage was recognized by vacuolated cytoplasm (Fig. 2B).

### Microscopic dermal structures of day 8 embryos using methylene blue and PAS

**Figure 3:**
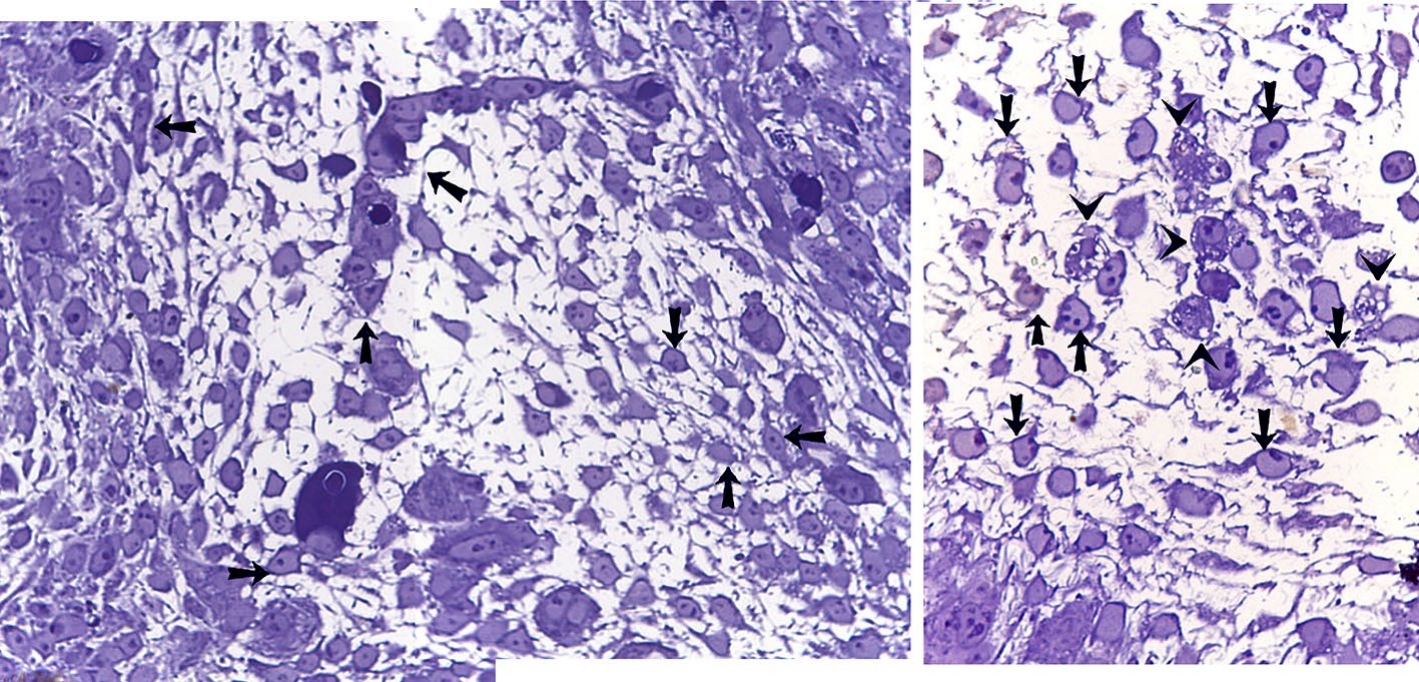
Microscopic dermal structures of day 8 embryos using methylene blue. Semithin section of skin embryos stained by methylene blue. (**A**): the dermis formed of a loose connective tissue. TCs (black arrows) located around the sprouting endothelial cells that lack the lumen. (**B**): The features of the principle type of interstitial cells (arrows) appear like TCs that had a cell body and cellular prolongations. Macrophage had vacuolated cytoplasm (arrowheads).

**Figure 4:**
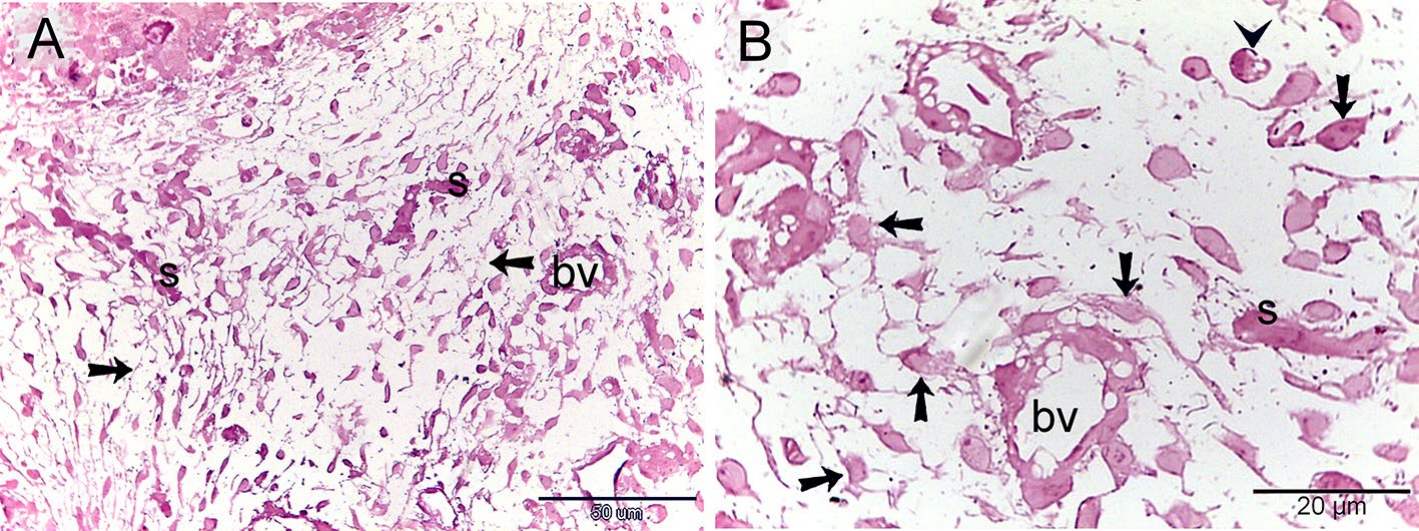
Microscopic dermal structures of day 8 embryos using PAS. Semithin section of skin embryos stained by PAS. (**A**, **B**): The morphology of main type of dermal cells (arrows) like TCs that had a cell body and cellular prolongations. Macrophage had vacuolated cytoplasm (arrowheads). TCs (black arrows) located around the sprouting endothelial cells that lack the lumen.

TCs were the prevalent interstitial cells. They had a cell body and cellular prolongations, TCs located around the sprouting endothelial cells that lack the lumen (Fig. 3A, Fig. 4A, B). TCs found around macrophages that had vacuolated cytoplasm (Figs. 3B, 4B).

### Ultrastructural organization of dermal TCs

**Figure 5:**
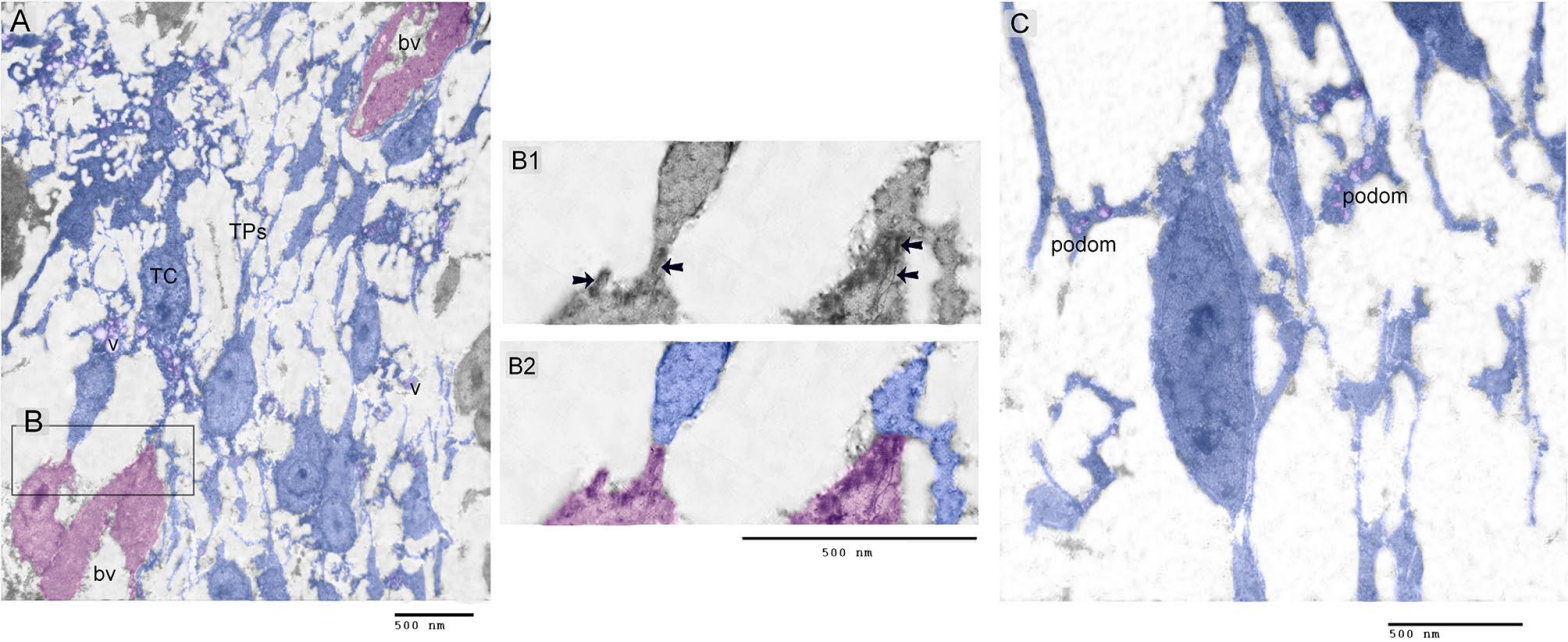
identification of dermal TCs using TEM (Transmission electron microscopy). (**A**): Colored ultrathin section. Telocytes (blue colored) formed a major continue of the interstitial tissue and established 3D network connected to each other by homocellular contact (double arrowhead). Telocytes were recognized by the unique TPs and podoms which rich in secretory vesicles (V). TCs established cellular contact (double arrows) with sprouting endothelial cells. TCs deformity and TPs were highly arborized (asterisk) with numerous podoms. Note endothelial cells (pink colored) and blood vessel (bv). (**B1**) (ultrathin section) and (**B2**) (Colored ultrathin section): showed areas of heterocellular of TPs with sprouting endothelial cells. Note localization of cytoskeletal filaments at the site of cell junction as well as pseudopodium (ps) that projecting form endothelial cells. (**C**): Colored ultrathin section of TC. TCs had 3D network that established by their TPs. Note podoms, secretory vesicles (V).

Telocytes formed the primary interstitial element and established a 3D network connected to each other by homocellular contact. Telocytes were distinguished by the unique TPs and podoms, which are rich in secretory vesicles. TCs establish cellular contact with sprouting endothelial cells. TCs deformity and TPs were highly arborized with numerous podoms. TCs formed heterocellular cells with sprouting endothelial cells. Cytoskeletal filaments identified at the site of cell junction with TCs as well as pseudopodium that project from endothelial cells (Fig. 5A–C).

### Immunohistochemical identification of TCs in the embryonic skin

**Figure 6:**
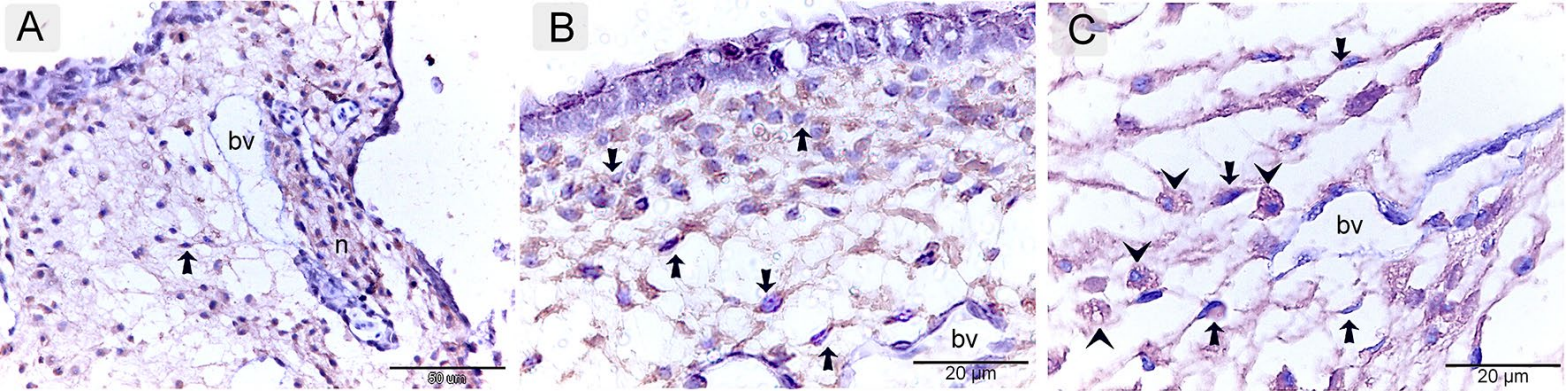
Immunohistochemical staining of the embryonic skin with CD34. Paraffin sections stained with CD34. (**A**–**C**): CD34-postive telocytes (TCs; arrows) forms the dominant dermal interstitial cell. note blood vessels (bv), dermal embryonic macrophage exhibited positive immune-reactivity for CD34.

**Figure 7:**
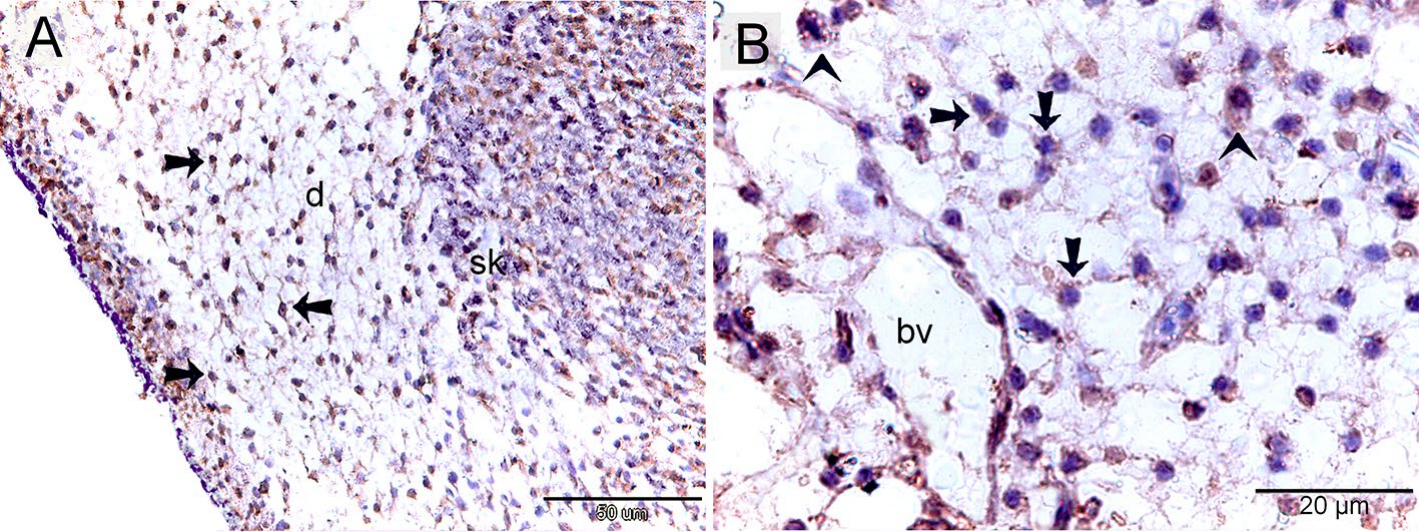
Immunohistochemical staining of the embryonic skin with VEGF. Paraffin sections stained with VEGF. (**A**, **B**): VEGF -postive telocytes (TCs; arrows) forms the main type of dermal interstitial cells. Note blood vessels (bv), dermal embryonic macrophage exhibited positive immune-reactivity for VEGF.

**Figure 8:**
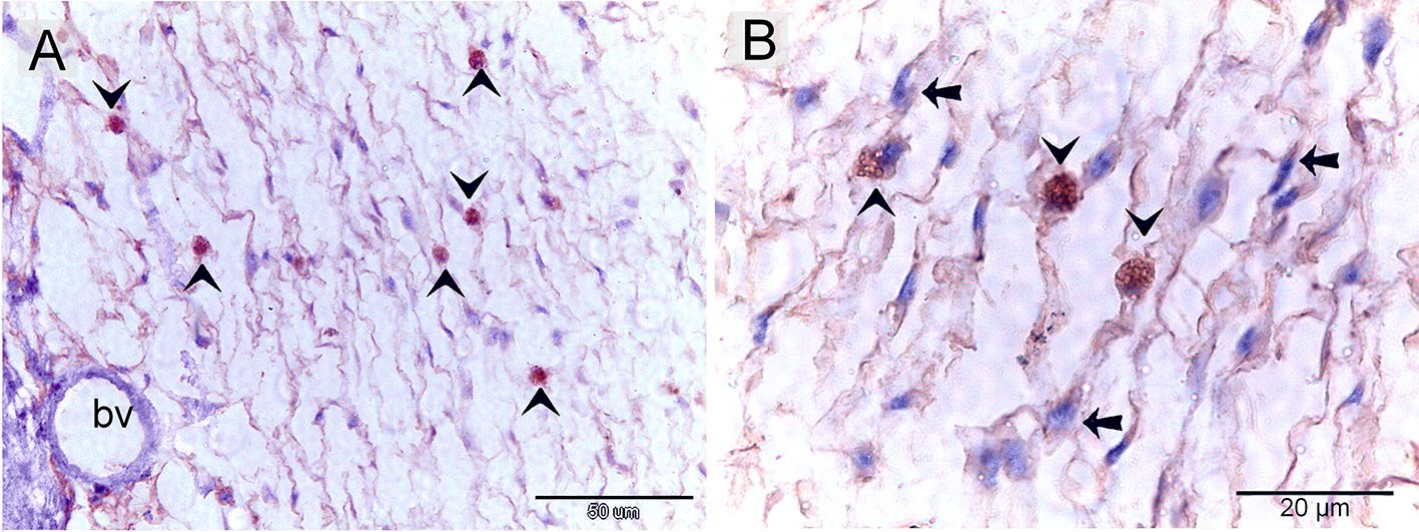
Immunohistochemical staining of the embryonic skin with CD117. Paraffin sections stained with CD117. (**A**, **B**): CD117-postive telocytes (TCs; arrows) constitute the prevalent interstitial cells of the dermis. Note blood vessels (bv), dermal embryonic macrophage exhibited positive immune-reactivity for CD117.

TCs expressed CD34 (Fig. 6A–C), VEGF (Vascular Endothelial Growth Factor) (Fig. 7A, B), and CD117 (C-Kit or c-kit receptor tyrosine kinase) (Fig. 8A, B). They represent the dominant dermal interstitial cells. They organized a 3D network enclosing other cells and structures located in the dermis, including growing blood vessels, nerve fibers, and macrophages (Figs. 6A–C, 7A, B, 8A,B).

### Proteolytic activity of TCs

**Figure 9:**
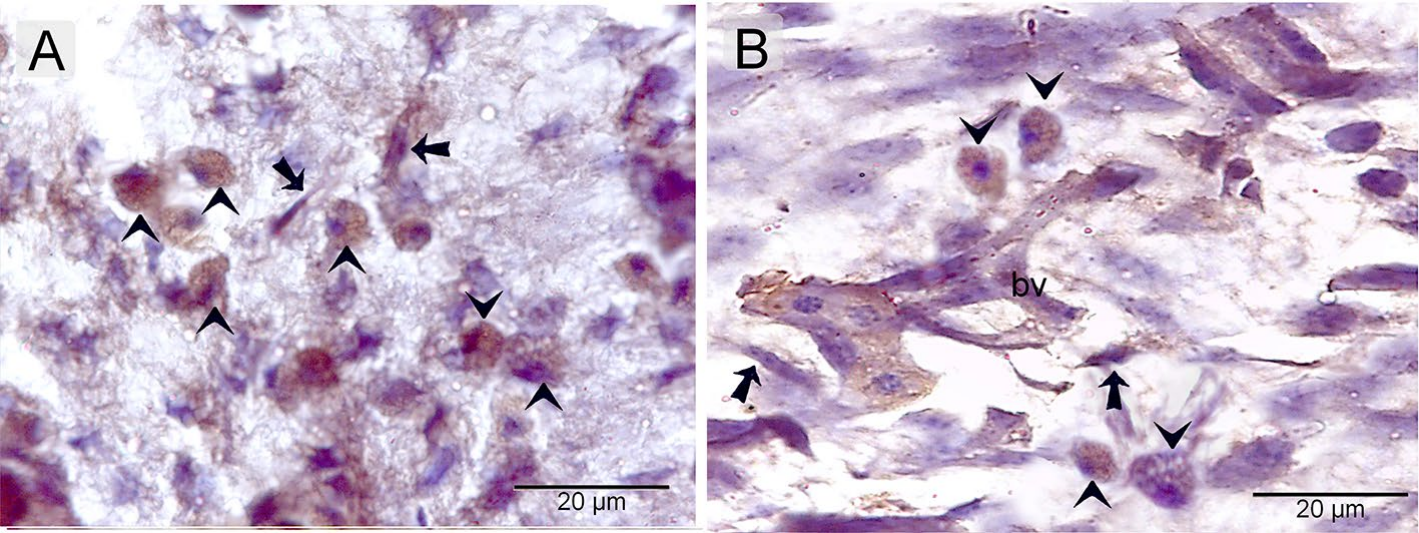
Immunohistochemical staining of the embryonic skin with MMP-9. Paraffin sections stained with MMP-9. (**A**, **B**): MMP-9-postive telocytes (TCs; arrows) located in the dermal tissue note blood vessels (bv), dermal embryonic macrophage exhibited positive immune-reactivity for MMP-9.

**Figure 10:**
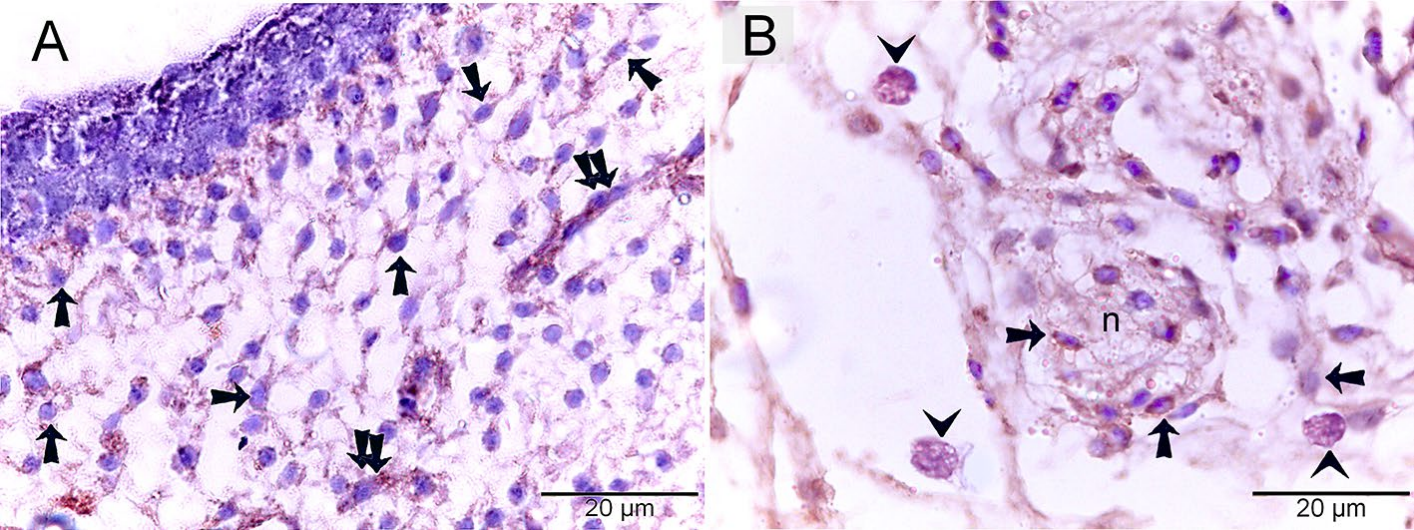
Immunohistochemical staining of the embryonic skin with CD68. Paraffin sections stained with CD68. (**A**): CD68-postive telocytes (TCs; arrows) were the chief type interstitial dermal cells. TCs located around the sprouting blood vessels (double arrows) that lack a lumen. (**B**): TCs (arrows) located around the nascent nerve fiber that recognized by loose organization neural ellements. Dermal embryonic macrophage exhibited strong immune-reactivity for CD68.

**Figure 11:**
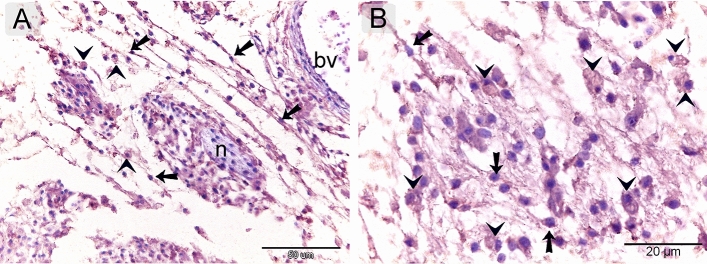
Immunohistochemical staining of the embryonic skin with CD21. Paraffin sections stained with CD21. (**A**, **B**): CD21-postive telocytes (TCs; arrows) constitute the principle type of interstitial cells of the dermal tissue. Note nerve (n) and blood vessels (bv). Dermal embryonic macrophage exhibited strong immune-reactivity for CD21.

Both TCs and macrophages expressed MMP-9 (matrix metalloproteinases-9) (Fig. 9A, B), CD68 (Fig. 10A, B), and CD21 (Fig. 11A,B). TCs are located around the sprouting blood vessels (Fig. 10A) as well as the nascent nerve fiber (Fig. 10B).

## Discussion

The current results demonstrate the immunological features and distribution of dermal telocytes in the early stages of embryonic development. Dermal embryonic TCs had phenotypic features similar to adult TCs^26^. They had a cell body and telopodes which distinguished by the unique podomeres and podoms. Secretory vesicles were abundant in podoms, and TCs released them into the extracellular matrix. They established a 3D network spread throughout the dermis. TCS connected to each other by homocellular and heterocellular contacts. Thus, TCs transmit signals through extracellular vesicle-mediated paracrine signaling or contact with neighboring cells.

Immunohistochemical techniques using CD34, CD117, and VEGF confirmed TC identification. TCs represent the major interstitial component in the dermal tissue. They established a 3D network enclosing other cells and structures in the dermis, including growing blood vessels, nerve fibers, and macrophages. It seems that TCs formed the interstitial infrastructure of the embryonic dermis. The organization of the TC network spreading through the dermal tissue may reveal the role of TCs in providing mechanical support and evidence for the 3D interstitial network of telocytes, which serves as a scaffold for organizing the architecture of the dermis before the development of dermal structures and cells.

In the current study, TCs exhibited proteolytic activity that expressed MMP-9, CD68, and CD21. MMP-9 helps migratory cells invade the tissue by clearing away extracellular matrix elements in their path and facilitating the development of new structures*.* MMP families, also known as matrixins, are crucial for tissue remodeling and development because they catalyze the breakdown of extracellular matrix components. MMP-9, also known as gelatinase B, is a metal-dependent endopeptidase that is involved in angiogenesis, immune cell migration, chemokine and cytokine activation, and the progression of cancer cells^20^. Among the ECM components on which MMP-9 can act proteolytically are collagen types IV, V, XIk', XIVl', elastin, aggrecan, link protein, decorin, laminins, entactin, SPARCq, myelin basic protein, 2Mn, 1Pli, IL-1j, and proTNF^21^.

The current study used specific phagocytic markers for the identification of macrophages. The expression of CD68 is specific for phagocytic cells, which have developed their endosomal-lysosomal systems. The expression of CD68, a member of the D scavenger receptor family, correlated with the maturation of late endosomes and lysosomes. Examples of phagocytes that carry CD68 in their granules^22^. Complement receptor 2, or CD21, is an endogenous ligand that reacts to complement component C3 fragments, low-affinity immunoglobulin (Ig)E receptor CD23, and type I cytokine interferon-alpha. CD21 is an essential component of activated B- and T-lymphocytes. Two innate immune receptors that CD21 interacts with are DNA-DNA complexes (chromatin) and interferon, an antiviral cytokine^23^**.**

Expression of VEGF by TCs reveals the angiogenic role of TCs during dermal development. This is supported by TEM observations. TCs establish cellular contact with sprouting endothelial cells. Cytoskeletal filaments identified at the site of cell junction with TCs as well as forming the pseudopodium core that project from endothelial cells. Pseudopodium formation is linked to the polymerization of the cortical actin^24^. Pseudopodium helps in cell movement and migration during neo-angiogenesis^25^.

Angiogenesis is the process by which blood vessels develop from the preexisting vasculature^26^. A variety of cell types controls a complex and dynamic angiogenic system, including TCs^27^ and macrophages ^28^. TCs express VEGF which promote formation of new vasculature^27^. TCs express VEGF, which promotes the formation of new vasculature. Macrophages may participate in angiogenesis via the secretion of various cytokines, growth factors, or ECM-modifying proteins^29^.

Telocytes influence other types of cells through cell contact or paracrine signaling. Proteins, receptors, bioactive lipids, nucleic acids, including mRNA, microRNA (miRNA), and non-coding RNAs, are all transported by secretory vesicles^30,31^**.** Telocytes produce compounds of different molecular weights, such as proteins or RNAs^32^. In addition, telocytes provide growth factors, chemo-attractants, cytokines/chemokines, macrophage inflammatory proteins 1 and 2 (MIP-1 and MIP-2), and monocyte chemoattractant protein 1 (MCP-1). Moreover, TCs regulate macrophage activity through paracrine signaling and hepatocyte growth factor (HGF)^33^. TCs also promote macrophage differentiation and phagocytosis and inhibit macrophage apoptosis via the activation of NF-κB^34^. This data reveals that TCs have a role in immunological regulation.

VEGF belongs to a family of growth factors derived from platelets. Some of the common roles of VEGF during embryonic development are angiogenesis^35^, vascular permeability^36^, and vascular integrity^37^.

Endothelial cells that migrate and proliferate in response to a VEGF gradient activate the VEGF receptor 2 (VEGFR2), which is present on their surface. Moreover, macrophages can secrete semaphorins, which provide "vascular guidance" for the endothelial cells. The following substances have been identified: TGF-transforming growth factor, TNF-tumor necrosis factor, uPA (urokinase plasminogen activator), VEGF (vascular endothelial growth factor), ECM (extracellular matrix), EGF epidermal growth factor, IL-1, IL-10, MMP matrix metalloproteinases, and SEMAs semaphorins^29^.

Tyrosine kinase CD117, also referred to as cKit, is expressed by a wide range of stromal cells. CD117 interacts with stem cell factor (SCF), a cytokine growth factor. SCF's cellular engagement of CD117 in germ and stem cells enhances distinct cytokine-dependent signaling pathways that promote a range of cell activities, such as survival, proliferation, differentiation, and migration^35,36^. For stem cells, including hematopoietic stem cells, epithelial stem cells, muscle satellite cells, interstitial cells, corneal keratinocytes, and vascular endothelial progenitors, transmembrane phosphoglycoprotein, or CD34, is a characteristic marker^38^.

TCs influence the activities of different types of dermal cells. They promote angiogenesis through the expression of VEGF and facilitate endothelial cell migration through the degradation of the extracellular matrix via MPP-9. Previous data support the regulatory function of the TCs and their role in the promotion of the formation of dermal structures. FGF signaling regulates fibroblast activation^39^. FGF expressed by TCs^40^. TCs also control macrophage activities^41^. TCs express TGFβ^42^ which promotes the thermogenic function of adipocyte^42^. The organization of TCs network extending throughout the dermal tissue enables TCs to reach the target cells and perform their function. Moreover, TCs 3D network acts as a scaffold to organize different dermal structures. Thus, TCs are a critical source of signaling molecules that regulate the activities of dermal cells and the formation of fibrous components and act as the biological and structural scaffold for the development of other dermal components.

## Conclusion

The majority of the findings that have been discussed now come from morphological analysis. This is an intentional choice because morphology provides a convincing approach for recognizing the functions of the various cell types seen in the embryonic dermis. Morphology can be used to recognize types of cells based on their distinctive characteristics and to identify the presence of cellular contact with other cells or with one another. These data can help understate the functional roles that can be inferred or speculated on with other techniques.

It was possible to definitively recognize the TCs and prove that they constitute a distinct population of interstitial cells using IHC and TEM. Moreover, intestinal TCs were distinguished from those found in other systems through the examination of their phenotypes. Moreover, the macrophages' shape and immunological nature enabled their description. Regarding morphology, the formation of the entity of blood vessels allows for the description of the endothelial cells.

From the available data, it appears that TCs and endothelial cells establish cell-to-cell contact and, likely, interplay in order to perform their functions in the embryonic dermis. On the other hand, the coexistence of macrophages within the 3D interstitial network established by TCs and the data presently discussed reveals interactions between TCs and macrophages^43^. In this relationship, the TCs often perform a role of guidance and nursing to ensure the macrophages' functioning.

Direct physical cell-to-cell interaction, which is likely to be accountable for facilitated the development of heterocellular junctions between TCs and endothelial cells the recently discovered, highly effective method of controlling endothelial cell activity and angiogenesis. TCs-educated endothelial cells might be a promising way to restore defective dermal structures, leading to enhanced treatment efficacy for dermal disorders.

The current study investigated the early structures of the embryonic dermis before the formation of the fibrous components and the appearance of the resident dermal cells. TCs formed a 3D network, which comprised the primary intestinal component of the early embryonic dermis. These data reveal that dermal TCs probably serve as the biological and structural scaffold for the development of the dermis. The current study sheds light on the dermal TCs that appeal to consider the probability that increasing the density or existence of dermal TCs may prove to be a highly desirable therapeutic approach for managing many skin disorders.

### The key findings

- TCs comprised the principle interstitial cells of the dermis
- TCs established homocellular and heterocellular contact with sprouting endothelial cells.
- Expression of VEGF by TCs indicate their role in angiogenesis.
- Expression of MPP-9 and CD68 reveal proteolytic activity of TCs
- Expression of CD21 of TCs suggesting their immune function.

## Material and methods

### Ethical approval

The National Ethics Committee of South Valley University and the veterinary authorities in Qena Province, Egypt, approved the methodology used in this study. "All procedures were carried out in compliance with the applicable policies and guidelines."

Fertilized quail (Coturnix japonica) eggs have been purchased from the Research Quail Farm, which is linked to the Department of Histology at the Faculty of Veterinary Medicine, South Valley University, Qena, Egypt. The incubation conditions for the fertilized eggs were 37.5 °C and 65% relative humidity. After the third day of incubation, the eggs were automatically turned every six hours. On the eighth day of incubation, fertilized eggs were collected and stored at − 20 °C for four hours before the collection of embryos. The embryos that seemed healthy were carefully extracted from the eggshells by opening the broad end of the shells. The abdominal skin was carefully excised before fixation. Skin samples were taken from early embryonic stages, where the growth and development of various interstitial constituents are *proceeding a*ccording to previous studies^27,44^. Three embryos were used for the histochemical and immunohistochemical procedures, and three more were used for TEM.

*Fixation*: All embryos used for light microscopic analysis were fixed immediately in 10% neutral buffered formalin and left for 30 min in Bouin's solution. After that, the fixed samples were dehydrated through ascending concentrations of ethanol and alcohol dehydration (70%, 80%, 90%, and 100%). The samples were then cleaned using methyl benzoate. After that, dehydrated samples were embedded in Paraplast (MilliporeSigma, St. Louis, MO, USA) and impregnated. The samples' paraffin-embedding processing times are listed in Table 1.

**Table 1:** The processing time of the samples in paraffin embedding techniques.

Age process	5d	8d	15d
1-Fixation			
A-NBF	8 h	13 h	24 h
B_Bouin’s solution	1/2 h	1/2 h	1/2 h
2-dehydration Alcohol 70%I	2 h	2 h	2 h
Alcohol 70%II	2 d	2d	2d
Alcohol 70%III Alcohol 80%	1 h	2 h	2 h
Alcohol 90%	1 h	2 h	1 h
Alcohol 100%	1/2 h	1/2 h	1/2 h
Alcohol 100%	1/2 h	1/2 h	1/2 h
3-clearing with methylebenzot			
MBI	1 h	1 h	1 h
MB II	12 h	12 h	12 h
MBIII	12 h	12 h	12 h
4-embedding in paraffin:			
P I	2 h	2 h	1/2 h
P II	2 h	2 h	1/2 h
PIII	**4 h**	**4 h**	1 h

NBF Neutral buffer formalin, h, Hours; d, Days; MB I, Methyl bonzoate1, MB II, Methyl benzoate II; PI, Paraffin I; P II, Paraffin II; P III, Paraffin III.

### I- Histological examination

Using a Leica RM2125 microtome (Leica Microsystems, Wetzlar, Germany), serial 5-µm transverse and longitudinal sections were cut, and to ensure dryness, the sections were stored in an incubator at 40 °C. Hematoxylin and eosin were used to stain the sections for a general histological examination^45^.

### II-Preparations of resin embedding samples for semi-thin sections

Karnovsky's fixative was used for the resin-embedding technique. The following is how the fixative was made: 30% distilled water, 50% glutaraldehyde, 25% paraformaldehyde, and 50 mL phosphate buffer are combined with 10 mL each. Samples were used from day 8 embryos. The skin on the neck was carefully removed and cut to a length of 2.0–3.0 mm. Overnight at 4 °C, Karnovsky fixative (Table 2).

**Table 2:** Components of the fixative.

Fixative	Components	Amount
Karnovsky Fixative	Paraformaldehyde, 25% freshly prepared	10 ml
	Glutaraldehyde 50%	10 ml
	Na-Phosphate buffer (0.1 M, pH 7.4)	50 ml
	Distilled water	30 ml
N a-Phosphate buffer (0.1 M, pH 7.4)	Solution A	
	Na2HPO4 2H2O	17.02 gm
	Distilled water	600 ml
	Solution B	
	NaH2PO4 H2	6 gm
	Distilled water	200 ml
	Using solution	
	Solution A	580 ml
	Solution B	219 ml
Citrate-buffer (pH 6.0)	Solution A	
	Citrate C6H8O7 H2O	21 g
	Distilled water	1 L
	Solution B	
	Sodium citrate Na3C6H5O7 2H2O	29.41 g
	Distilled water	1 L
	Using solution	
	Solution A	9 ml
	Solution B	41 ml
	Distilled water	Add 500 ml

The samples were postfixed with osmium tetroxide, embedded in resin, cleaned, and crystallized in an oven set to 60 °C. They were also impregnated with a pure resin/alcohol mixture. Propylene oxide (Merck, Darmstadt, Germany) was used to embed the resin. Next, for approximately half an hour, a 1:1 mixture of epoxy resin and propylene oxide was used, and lastly, the epoxy resin mix was used for three hours. The epoxy resin mixture was prepared by combining 5 mL of Araldite (Huntsman Advanced Materials, The Woodlands, TX, USA) and 5 mL of EMbed 812 (Polysciences Europe GmbH, Eppelheim, Germany) with 12 mL of dodecenylsuccinic anhydride (DSAA). To polymerize the samples, the epoxy resin mixture was heated to 60 °C after the samples were embedded in it. Then, an accelerator (2,4,6-Tris[dimethylaminomethyl]phenol; 1.5%) was added to the mixture. Three days of incubation were given to the blocks: one at 60 °C, one at 70 °C, and one at 75 °C^45^.

Semithin sections were cut at 1 μm using an ultramicrotome Ultracut E (Reichert-Leica, Germany) and then stained with toluidine blue and Periodic acid-Schiff (PAS)^27,44,46–51^. After dissolving the resin in a saturated alcoholic solution of sodium hydroxide, semithin sections were stained. A Canon PowerShot A95 digital camera and a Leitz Dialux 20 microscope were used to examine the stained sections.

### III- Immunohistochemistry staining

#### Immunohistochemistry staining procedures for CD34, CD117, CD68, and MMP-9

The anti-polyvalent, horseradish peroxidase/3,3´-diaminobenzidine (DAB), Ready-to-Use reagent (Thermo Fisher Scientific, Waltham, MA, USA) was utilized in compliance with the manufacturer's instructions to accomplish antigen localization through the avidin–biotin complex technique in the Lab Vision Ultra Vision Detection System^52^.

The process was, in short, as follows^53–55^: The 5-µm-thick paraffin sections were rinsed three times with a pH 7.4 phosphate-buffered solution (PBS) for five minutes each. After that, they were dewaxed with xylene and rehydrated with progressively higher ethanol and alcohol grades. To prevent endogenous peroxidase activity, the slices were kept in blocks of hydrogen peroxide at room temperature. The pieces were then washed for an additional ten minutes under running tap water. To enhance antigen retrieval, the slides were treated for 20 min at 95–98 °C in a water bath using a 10-mmol sodium citrate buffer (pH 6.0; Table 2). The slides were then allowed to cool for twenty minutes at room temperature before being cleaned with PBS three times for five minutes apiece (pH 7.4). Nonspecific background staining was blocked for five minutes at room temperature using Thermo Fisher Scientific's Ultra V Block. By limiting the staining time to no more than ten minutes, this was done to prevent staining the artifact. Following the application of the primary antibody (Table 3) to the sections for an overnight period at 4 °C, the sections were washed three times for five minutes each using PBS (pH 7.4). The secondary antibody (Table 3) was applied to the sections and allowed to sit at room temperature for ten minutes. The sections were then treated with three 5-min PBS washes (pH 7.4) and incubated with a streptavidin-peroxidase complex (Thermo Fisher Scientific UK and Lab Vision Corporation) for ten minutes at room temperature. To see the bound antibodies, two milliliters of DAB plus substrate and one drop of DAB plus chromogen were combined, applied to the sections, and allowed to sit at room temperature for five minutes. The incubation process was conducted in a humid chamber. Harris haematoxylin, the counterstain, was applied and left for 30 s. Following a five-minute dehydration in 100% ethanol twice, the sections were cleaned in xylene and coated in DPX (dibutylphthalate polystyrene xylene) mounting solution. To examine the immunohistochemistry staining, we used a Leitz Dialux 20 microscope (Leitz GmbH, Oberkochen, Germany) and a Canon PowerShot A95 digital camera (Canon Inc., Tokyo, Japan).

**Table 3:** Identity, sources, and working dilution of antibodies used in Immunohistochemical studies.

	Supplier	Origin	Dilution	Incubation	Antigen retrieval	Biotinylated secondary antibodySupplier
CD117 (Anti –c-Kit)	Genremed Biotechnologies, Inc, South San Francisco, USA	Rabbit polyclonal	1:50	1 h at room temperature	boiling in citrate buffer (pH 6.0), 20 min	Goat anti polyvalent
Anti- MPP9	Thermo Fischer Scientific, Lab vision Corporation, Fremont, USA	Mouse (mc, Ab-1) Clone D(33)376 Rabbit polyclonal	1:30	Over night	boiling in citrate buffer (pH 6.0), 20 min	Goat anti polyvalent
CD34	MOUSE ANTI CHICKEN CD34 (Bio rad)	MOUSE ANTI CHICKEN CD34 Monoclonal Antibody (Clone: AV138) (Cat.no MBS224490)	1:100	Over night	boiling in citrate buffer (pH 6.0), 20 min	Goat anti-Mouse IgG (H + L) Secondary Antibody Catalog # 31,569 Dilution ; 1:100 One hour at room temperature
CD68 (Macrophage Marker) Ab-3 (Clone KP1)	Mouse Anti-CD68 Thermo Fisher Scientific Lab Vision Corporation, Fremont, USA	Mouse Monoclonal Antibody Cat. #MS-397-R7	1:100	Over night	boiling in citrate buffer (pH 6.0), 20 min	
VEGF	Rabbit anti -VEGF (Invitrogen by Thermo Fisher Scientific Waltham, MA, USA))	Rabbit VEGF Polyclonal Antibody (clone: RB-222- P0) (Cat.no PA1-21,796)	1:100	Over night	boiling in citrate buffer (pH 6.0), 20 min	Goat anti-rabbit secondary antibody (cat. no. K4003, EN Vision + TM System Horseradish Peroxidase Labelled Polymer; Dako) Ready to use 30 min at room temperature
CD21	Rabbit Anti- CD21 antibody (Abcam)	Rabbit Anti-CD21 antibody monoclonal SP186](ab227662)	1:100	Over night	boiling in citrate buffer (pH 6.0), 20 min	

Antibodies used that showed reactivity in avian species.

#### Immunohistochemical procedures for vascular endothelial growth factor (VEGF)

The Dako EN Vision + Single Reagent (HRP. Mouse; Agilent Technologies, Inc., Santa Clara, CA, USA) was employed for the two-step immunohistochemical staining procedure^56^. We used the staining technique created by Abdo and colleagues. To sum up, five-micrometer-thick paraffin-embedded sections were dewaxed, rehydrated, and rinsed three times with PBS (pH 7.4), each time lasting five minutes. To suppress endogenous peroxidase activity, methanol was treated with drops of 3% hydrogen peroxide and allowed to sit at room temperature for 20 min. Then, it was washed under running water for an additional 10 min. In order to extract antigen, slides were immersed in a 10-mm sodium citrate buffer (pH 6.0; Table 2) and heated to a temperature of 95–98 °C in a tap water bath for 20 min. The slides were then allowed to cool for a further twenty minutes at room temperature. After that, the sections were cleaned three times with PBS (pH 7.4) for five minutes each. Drops of Dako Protein Block (Agilent Technologies, Inc.) were applied to each segment and left to sit at room temperature for five minutes in order to prevent nonspecific background staining. It should be noted that prolonged blocking may result in less staining than intended. Sections were then subjected to the primary antibody treatment (one of the antibody types utilized in a recent article demonstrated reactivity in avian species)^55^. The names, sources, and working dilutions of every antibody used in immunohistochemistry research are listed in Table 3. After the incubation period, slides were cleaned three times for five minutes each using PBS (pH 7.4) after being incubated with the secondary antibody for thirty minutes at room temperature (Table 3). The slides were again treated with DAB and substrate-chromogen for five to ten minutes at room temperature after three five-minute rinses in PBS (pH 7.4). This leads to the antigen location producing a brown precipitate. The sections were counterstained for 30 s with Harris hemoxoxylin. The sections underwent two rounds of dehydration, lasting five minutes each in 90% and 100% ethanol, after being cleaned in xylene and coated with DPX. As before, we evaluated immunohistochemistry staining using the Leitz Dialux 20 microscope and the Canon PowerShot A95 digital camera. We used a primary antibody-free version of the same protocol to generate negative control samples.

### Supplementary Information

Supplementary Information.

## Data Availability

The data sets collected and/or analyzed during the current study are available from the corresponding authors on reasonable request.
