# Supplementary material for: Immunohistochemical-properties of the dermal embryonic telocytes
Source: Sci Rep. 2024 Jun 17;14:13899. doi: 10.1038/s41598-024-63802-5 (PMC11183069; doi:10.1038/s41598-024-63802-5)
Supplement: Supplementary file 1 — Supplementary Information. [file 41598_2024_63802_MOESM1_ESM.docx]

Suplmentary materials

| Quail birds fed on “**Wild Delight Dove & Quail Food”,** Brand: Wild Delight. It is a special blend of premium seeds and grain formulated for quail and other birds. It contains Crude Protein (Min) 6.00%, Crude Fat (Min) 2.00% and Crude Fiber (Max) 10.00%. it composed of Millet, Milo, Wheat, Popcorn, Oat Groats, Safflower Seed, Sunflower Kernels, Sunflower Seed. |  |
| --- | --- |

Incubation period of the Japanese quail is 17 days
